# *"Pundits Are Saying This Is ‘Anti-poor’"*: Competing Framing Strategies for Child Road Safety Policy in the Philippines

**DOI:** 10.34172/ijhpm.8585

**Published:** 2025-07-21

**Authors:** Sarah N. Champagne, Adam D. Koon, Allan R. Ulitin, Haidee A. Valverde, Lamisa Ashraf, Ma-Ann M. Zarsuelo, Connie Hoe, Hilton Y. Lam, Abdulgafoor M. Bachani

**Affiliations:** ^1^Johns Hopkins International Injury Research Unit, Health Systems Program, Department of International Health, Johns Hopkins Bloomberg School of Public Health, Baltimore, MD, USA.; ^2^Department of Global Health, School of Health, Georgetown University, Washington, DC, USA.; ^3^Institute of Health Policy and Development Studies, National Institutes of Health, University of the Philippines Manila, Manila, Philippines.; ^4^Heidelberg Institute of Global Health, Faculty of Medicine and University Hospital, Heidelberg University, Heidelberg, Germany.

**Keywords:** Framing Theory, Policy Analysis, Child Health, Injury, Child Restraint Systems, Philippines

## Abstract

**Background:**

Child restraint systems (CRS) can lead to a 60% reduction in child deaths, yet few low- and middle-income countries (LMICs) have comprehensive policies to enforce best practice standards. In 2019, the Philippines established such a policy: the Child Safety in Motor Vehicles (CSMV) Act.

**Methods:**

Drawing on framing theory, this study aims to understand the social dimensions of policy change to identify the Act’s origins, design, and implementation. Three sources of data – 25 articles, 27 key-informant interviews, and field notes – were collected and thematically analysed.

**Results:**

We present the findings according to two features of the framing process: storytelling and naming. The policy process can be sharply distinguished into two sections: the Act’s passage into law (which was swift and successful) and its implementation (which to date has not been). The Act’s implementation was stymied by three overarching frames – that it is "anti-poor," "unnecessary," and a "strategic political distraction." A media backlash at the time of implementation solidified these frames, leading President Duterte to indefinitely defer enforcement of the Act.

**Conclusion:**

The CSMV Act emphasises that passing a law is insufficient. The trajectory of the act highlights the combined importance of (a) the framing of policy, (b) framing processes operate throughout a policy’s lifecycle, and (c) the media in creating a narrative. Our findings offer valuable insights for other LMICs implementing evidence-based road safety measures, suggesting that successful implementation requires not only strong legislation but also strategic communication and frame management throughout the policy process. Understanding framing dynamics can help policy-makers anticipate and address potential resistance to life-saving public health interventions.

## Background

**Key Messages**

**Implications for policy makers**

- While public health policy, for example, road safety laws such as the Philippines’ Child Safety in Motor Vehicles (CSMV) Act, may be effectively passed into law without public support, early public support is important to ensure effective implementation.
- The way a policy is framed – that is, the complex ways in which actors make sense of policy, use language to emphasise specific features of policy, and tell persuasive stories about policy – plays an important role in the political life and success of a policy.
- The media is a central broker in the framing of policy due to its ability to substantively establish narratives, for instance, the Philippines’ CSMV Act was fully legislated when a traffic official’s insensitive remark led to a media backlash establishing the law as “anti-poor.” This unfavourable framing propagated by the media was sufficient to cause the president to indefinitely ban enforcement of the CSMV Act without the legal backing to do so.

**Implications for the public**

Laws enforcing the proper use of child restraint systems (CRS), such as car seats, can save lives. Proper use of CRS can lead to a 60% reduction in child road traffic deaths, especially among children under 4 years old. However, as the case in the Philippines demonstrates, passing even a strong CRS law is not guaranteed to be effective without public buy-in. Following a media backlash, predominantly stressing that the law was not sensitive enough to the financial burdens of mandatory CRS use, the then President halted the enforcement of the act. How people make sense of a piece of legislation—how it is framed—matters and laws need to be sensitive to the views and concerns of the general public.

Globally, 1.35 million deaths are caused each year by road traffic crashes.^1^ Road traffic injuries are the eighth leading cause of global deaths and years of life lost, and this burden is projected to increase.^1^ Moreover, these trends are particularly acute in low- and middle-income countries (LMICs), which have road traffic death rates that are three times as high as high-income countries (HICs).^1^ In fact, 90% of global road traffic deaths occur in LMICs, despite having only about half of the world’s registered vehicles.^1^

 In response, LMICs are increasingly recognising the magnitude of the problem and developing plans to address road safety. In the Philippines, for example, an estimated 13 017 deaths were attributable to road traffic crashes in 2019.^2^ These trends are increasing, and road traffic injuries alone cost about 4.1% of the country’s gross domestic product.^3^ As a result, the Philippines developed a Road Safety Action Plan 2017-2022 to envision zero road traffic deaths, and a reduction of at least 20% by 2022.^4^

 Establishing and enforcing national child restraint systems (CRS) regulations can facilitate safety among road users by reducing the burden of disease among children.^5^ CRS can lead to a 60% reduction in deaths, with the greatest benefits accruing to younger children (under four years of age).^6,7^ For older children (8-12 years of age), booster seat use has been shown to reduce injury by 19% compared to seatbelts alone.^1^ However, national legislation and regulatory oversight are needed to facilitate and improve the adoption of CRS use by road users.^1,5,8,9^ Evidence-based public health legislation, inclusive of road safety legislation, has beneficial health impacts.^10^ To improve compliance and hence the positive health impacts, laws should be combined with swift, frequent, sustained, random, and widespread police enforcement to increase the perceived risk of being caught and reprimanded.^1^

 As of 2017, CRS legislation has been passed in 84 countries, 33 are considered to meet best practice criteria – of which four belong to LMICs.^1,5^ The United Nations’ (UN) Regulation Number 129 sets forward robust uniform provisions for CRS regulations, though CRS legislation can broadly be considered best practice if it is in place at the national level, is based on age, weight, and/or height factors, and restricts child front seat use below a certain age and/or height.^5,11^

 As of February 22, 2019, the Philippines has established a best practice CRS law in accordance with UN regulations, known as The Child Safety in Motor Vehicles (CSMV) Act, Republic Act (RA) No. 11229 of the Philippines. However, little is known about the law’s origins, design, or implementation. Understanding this is important for similar road safety legislation in the Philippines, as well as efforts to enhance regulatory oversight of CRS in other LMICs (Box 1).

**Box 1. Summary of the Child Safety in Motor Vehicles Act of the Philippines**

The CSMV Act corresponds to the UN Standards (UN Regulation No. 129). It includes three broad mandates: (1) Children 12 years and below must sit in rear seats of PMVs, unless they are taller than 150 cm, (2) A study on child safety in PUVs to be conducted by the Department of Transportation, and (3) The mandatory use of CRS in PMVs for children 12 and below, unless (*a*) they are over 150 cm in height, (*b*) are driving during a medical emergency, or (*c*) the child has a medical or developmental condition.

CRS must meet the standards set forward in UN Regulations 44 and 129, including its evolving standards and may not be marketed if expired. Under these mandates, manufacturers, importers, and vendors must obtain or ensure the CRS has the appropriate certifications to demonstrate the product has passed the Act’s standards. The CSMV Act stipulates drivers who do not observe the CRS or rear seat directives *“shall be fined one thousand pesos (PhP 1000.00) *[approximately US$ 20.00 as of the year 2021]* for the first offense; Two thousand pesos (PhP 2000.00) for the second offense; Five thousand pesos (PhP 5000.00) and suspension of the driver’s license for a period of one year for the third and succeeding offenses.”*^12^ Manufacturers, importers, and vendors of sub-standard, or expired CRS will face a penalty between fifty thousand and one hundred thousand pesos (*PhP 50, 000.00– PhP 100 000.00*) per CRS. Similarly, forgery of CRS certification will incur the same penalties.

----------------

Abbreviations: CSMV, Child Safety in Motor Vehicles; UN, United Nations; CRS, Child restraint systems; PMVs, private motor vehicles; PUVs, public utility vehicles.

The social life of policy designated for public health and injury prevention can be tied to a larger body of work on agenda-setting. In this research tradition, interests, institutions, and ideas converge to define problems, shape policy preferences, and construct target populations, among other things.^13^ Scholars studying these phenomena recognise the centrality of ideas in linking action to deeper values.^14,15^ This includes seminal sociological work on drinking and driving as well as more recent scholarship on political prioritisation for road safety in LMICs.^16-18^ Yet, it remains unclear how specific road safety policy is designed, legislated, and enacted in many LMICs.

 Frame analysis is an increasingly popular way of analysing the role of ideas in the policy process. There is no single approach to framing research, rather scholars from different disciplines have deployed diverse methods to explore a variety of health issues.^19^ Researchers from Critical Policy Studies have sought to advance framing theory by shifting the analytical focus from the frames themselves to the process of framing.^20,21^ This requires understanding the “what” as well as the “how” of framing in specific policy contexts. Entities that are framed in policy (the “what”) include: (1) actors’ identities and relationships, (2) substantive content/issues of the policy and (3) the interactive process.^22^ The “how” includes: (1) sensemaking, (2) naming, and (3) storytelling.^21^ While our study is focused on the “how” we locate elements of the “what” as constituents throughout storytelling before turning to naming. We propose that framing is a useful heuristic for understanding the social dimensions of policy change. This moves beyond static characterisations of frames that overemphasise structural explanations of policy change, instead situating agency within a political context. Moreover, it more clearly adds a discursive basis to recent explanations of policy entrepreneurship, and in a relatively unexplored policy domain (eg, road safety). In this way, we seek to understand the complex ways in which actors tell persuasive stories about policy, and use language to emphasise specific features of policy.^21,23^

 Despite its importance, little is known about the framing of road safety policy, particularly in LMICs. For these reasons, we conducted framing research on the CSMV Act of the Philippines to understand how it came to be a priority within the Philippines, the political process by which it was passed into RA No. 11229, and efforts to implement its provisions.

## Methods

 Three sources of data were used in this study. First, nine peer-reviewed articles specific to road safety in the Philippines and 16 pieces of grey literature (N = 25), including organisational and governmental reports were identified via hand searching and stakeholder recommendations. Specifically, authors asked informants to share any CSMV Act related documents and searched through PubMed, Google Scholar, and identified government and non-profit websites for literature related to the need for, passing of, or implementation of a CRS law in the Philippines. The authors also searched through the references of the identified documents for subsequent pertinent literature. Second, semi-structured key informant interviews were conducted with stakeholders and officials involved in the policy passage and implementation of the CSMV Act, including individuals involved in the coordination, advocacy, drafting, legislation, communication, enforcement, and education surrounding the Act. Participants were selected through purposive sampling to ensure representation across sectors involved in different aspects of the CSMV Act’s development and implementation, supplemented by snowball sampling as interviewees identified additional key stakeholders. Finally, field notes were recorded to capture the setting, flow, tone, and interviewees’ mannerisms throughout the interview. Including field notes as a data source allows us to capture both descriptive observations and reflective interpretations towards a richer understanding of the policy process.^24^

 A total of 27 key informant interviews were conducted from April–July 2021. Due to COVID-19 restrictions, all interviews were held remotely via Zoom. Interviews were led by SNC and were conducted in English with assistance and spot translation provided by ARU and/or HAV. In accordance with the Philippines’ National Ethics Committee’s regulations, spot translation was available should participants wish to express themselves in Filipino. Interviews lasted between 43 and 82 minutes, with an average of 61 minutes per interview.

 Many key informants who were both invited and agreed to participate in interviews were members of domestic non-governmental organizations (NGOs). This was consistent with findings from the document review, interviews, and snowball sampling that positioned NGOs as central to the passage and implementation of the CSMV Act.^25^ The complete breakdown of key informants can be found in Table 1. Saturation was achieved after 27 interviews.

**Table 1:** Key Informants Invited and Interviewed

**Sector**	**Interviewed**	**No Answer**	**Declined**	**Total Contacted**
Domestic NGOs	15	0	5	20
Legislative	3	4	0	7
Government	4	3	4	11
International NGOs/organizations	4	0	0	4
Academic	1	1	0	2
Private sector	0	2	0	2
**Total**	**27**	**10**	**9**	**46**

Abbreviation: NGOs, non-governmental organizations.

 All interviews were recorded, transcribed, thematically coded, and analysed using NVivo qualitative software. Peer debriefing was conducted immediately post-interview by the interviewer and the assisting interviewer(s), as well as periodically with ADK and the larger study team. Transcriptions were reviewed for accuracy by SNC and ARU. A codebook was developed using a combination of inductive and deductive methods. Ten overarching codes were employed: policy setting, Act’s features, merits and shortcomings, policy design, legislative process, implementation, public support, conflict, framing devices, and other, each with their respective inductive sub-codes.

## Results

 Drawing on our analysis of documents, key informant interviews, and field notes, we present the CSMV Act according to two features of the framing process: (1) storytelling: as a chronology of successful policy advocacy and incomplete implementation and (2) naming: using discursive frames to characterise the legislation.

###  Storytelling: Narratives of Policy Success and Implementation Shortfalls

 To begin, we will paint a picture of the broader narrative that explains the policy’s trajectory over time. This narrative reveals how the framing processes evolved throughout the different phases of the policy’s lifecycle, from successful advocacy and legislation to problematic implementation.

 As shown in Figure, the CSMV Act evolved over five years in the Philippines. An interesting feature of this timeline, which we describe in greater detail below, is the vast discrepancy between the design of the Act and the implementation of its provisions. Narratives such as this position a sequence of events as plausible explanations for how actors frame and reframe phenomena in the policy process.

**Figure:**
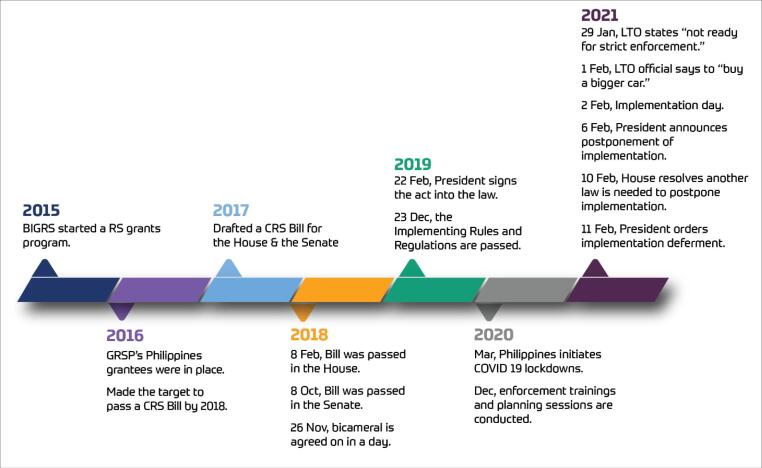
Timeline of the Child Safety in Motor Vehicles Act of the Philippines. Abbreviations: BIGRS, Bloomberg Philanthropies Initiative for Global Road Safety; GRSP, Global Road Safety Partnership; CRS, child restraint systems; LTO, Land Transport Office.

####  Act I: Actors, Advocacy and Agenda Setting

 In 2015, the Global Road Safety Partnership (GRSP) began the quest to support the passage of a piece of legislation that would address a behavioural risk factor for road safety in the Philippines, funded by Bloomberg Philanthropies as a part of the Bloomberg Philanthropies Initiative for Global Road Safety (BIGRS). The Philippines already had legislation around helmet use, drink driving, seatbelt use, and speeding, however, CRS use remained a “*main gap*” in road safety legislation.^26-29^ While an existing seat belt law in the Philippines included a clause that could allow the introduction of a CRS mandate, this was never actualised.^27^

 The GRSP established a call for grantees to provide funds to local NGOs and not-for-profit organisations that could drive the passage of a CRS policy. Subsequently, a coalition for the passage of a CRS Act was founded comprising members of many organisations listed in Table 2. The Global Health Advocacy Incubator, another member of the BIGRS, established a “country coordinator,” an on-the-ground official who organised the coalition by, for example, supporting logistically by setting up meetings and creating agendas. The coalition targeted legislation for CRS in the Philippines to be passed in 2018.

**Table 2:** Key Actors Involved in the Child Safety in Motor Vehicles Act of the Philippines

**Organisation Type**	**Organisation Name**
Domestic NGOs	ImagineLaw Inc. Initiatives for Dialogue and Empowerment through Alternative Legal Services VERA Files Buckle Up Kids Safe Kids Philippines New Vois Association of the Philippines Peace and Conflict Journalism Network Automobile Association Philippines Legal Engagement Advocating Development and Reform Center for Policy Studies and Advocacy on Sustainable Development
Legislative	Committee on Public Services (Subcommittee on Special Protection of Child Passengers) Committee on Women, Children, Family Relations and Gender Equality
Government	Department of Transportation Land Transport Office Philippine National Police Department of Trade and Industry Department of Health
International NGOs/organisation	Global Health Advocacy Incubator Philippines Red Cross World Health Organization Philippines United Nations Children’s Fund Vital Strategies Global Road Safety Partnership Bloomberg Philanthropies
Academic	Ateneo de Manila University School of Government

Abbreviation: NGOs, non-governmental organizations.

 Following American colonial rule, the Philippines’ political system has been based on the American model.^30^ For a policy to become legislation, it must be passed by the House of Representatives, by the Senate, and finally, the President can weigh in on the decision by signing, vetoing, or allowing the policy to lapse into legislation following three months of no presidential input. An early action item of the coalition was to identify policy champions in both branches of government (the House and the Senate) who could sponsor the CRS Bill. These politicians were identified largely based on their past support for road safety legislation and were recruited or lobbied to take on the passage of this Act. Legislative, government, domestic NGO, and international organisation interview respondents understood this coalition to be instrumental in the passage of what became the CSMV Act, though awareness of CRS and its importance was originally low among policy stakeholders.

 “*Political engagement right off the beginning went very smooth to start the process […] *[but] *we found out that there is hardly any awareness about child restraint systems. Like not among policymakers, not among [the] public*” (ID [identification] 24, International Organisation [sectoral classification of the informant]).

####  Act II: Legislation

 The legislative process to establish a CRS law faced relatively minor opposition and delays. House of Representative member Matt Jocabe argued that the Act is anti-poor because CRS are expensive and that families of lower socio-economic means cannot afford them. Furthermore, per Jocabe, the new CRS law would violate the equal protection clause of the Philippine Constitution because public transportation riders were not included. But this argument did not gain traction at the legislative stage.

 The process of establishing the final parameters of the law was broadly described by key informants across sectors as a “*compromise*” or achieving “*consensus*.” The substantive features of the legislation are consistent with global standards and informed by locally derived evidence. The GRSP grantees, led by Imagine Law, drafted the CRS Bills for the House and the Senate, with expert guidance provided by the GRSP and the World Health Organization (WHO) Philippines. The Bills relied heavily on a study commissioned by the WHO Western Pacific Regional Office titled: “A Baseline Study on the Availability, Affordability and Acceptability of Child Restraints in the Philippines.”^31,32^ The study was used to help adapt the UN’s best practice standards to be appropriate for the local context of the Philippines. Overall, there was very little disagreement about the Bill’s standards and requirements. Some small points of contention were: if CRS expiration dates should be considered; whether transport network vehicles (such as Grab taxis) and public utility vehicles (PUVs) (such as Jeepneys) should be included; the appropriate age cut-off for CRS; and whether the driver or the guardian should be liable in the case of improper CRS use. In the end, CRS expiration dates were included, CRS were not required in transport network vehicles and PUVs (however, the Act included the mandate of a study looking into the feasibility of requiring CRS use in PUVs), the age cut-off for CRS use was decided to be 12 years old, and the driver was made liable for CRS neglect or misuse. Key informants interviewed across sectors did not view these disagreements as fundamental challenges to the policy’s legitimacy but as technical details to be resolved through expert consultation and achieving consensus, reflecting a shared understanding that the policy’s basic premises were sound.

 Despite the anti-poor framing, and with support from technical experts and momentum from donor-financed actors, the process of drafting legislation can be widely appreciated as expedient. Both the House of Representatives and the Senate Bills were drafted by the same NGOs, resulting in few differences between the two Bills. Accordingly, when both Bills were passed, they were reportedly easily adapted and adopted to become the CSMV Act by legislative informants. This is also evident from the process only requiring a single day-long bicameral meeting.^33^ Nevertheless, some key stakeholders, particularly from domestic NGOs, considered the Bill to be “*held up*” in the Senate for a period in 2018. In the interim between the drafting and passage, other road safety activities, namely the GRSP’s Asia Pacific Regional Conference held in the Philippines and the Global Road Safety Leadership Course, another output of the BIGRS, brought together several key actors involved in advancing the Act. According to these informants, such parallel efforts helped sustain momentum and continued to motivate stakeholders working toward the passage of the CRS Bill. CRS road safety actors would similarly follow up with each other and outside government officials regularly via Viber groups and other forms of communication to keep up progress towards the successful passage of this Bill. Overall, the passage of the CSMV Act, a completely new bill, was executed in a little over two years, which, government and legislative study participants agreed, was unusually quick.

 The CSMV Bill was eventually signed into law by President Rodrigo Duterte on February 22, 2019, 89 days after the Bicameral Conference Committee on the Disagreeing Provisions of Senate Bill No. 1971 and House Bill No. 6938.^12,33^ As, in the Philippines, a President has three months (90 days) to sign or veto a bill before it lapses into a piece of legislation, select key informants from varying sectors believed that President Duterte delayed signing the Bill to ensure that it was “*uncontroversial*.” The strong support the CSMV Act received in the House and the Senate, combined with little industry, media, or public attention at the time, was believed by NGO and government informants to give the President confidence to pass the legislation. By signing the Bill, informants across sectors reported that President Duterte was showing his support for a “*good law”* – a law that saves the lives of children, has minimal impact on public funds, and does not upset his constituents.

 “*What the President was waiting for, was a public outcry against the law. I mean against the enrolled Bill that, and apart from public outcry, possible public outcry, should he pass the law” *(ID 04, Domestic NGO).

####  Act III: Implementation and Public Awareness

 At the time the CSMV Act was passed the Philippines did not yet have sufficient social infrastructure—governmental capacity and expertise—to robustly support the law. A major step towards achieving such social infrastructure was tasked to the Department of Transportation whose role was to create the Implementing Rules and Regulations (IRR) of RA No. 11229. These rules establish the mechanisms to ensure compliance with the Act, including public awareness strategies, access to CRS, and enforcement procedures. The IRR were passed later that year on December 23rd.^34^

 As public awareness of the law was very low come the end of 2019, and to ensure “*implementability*,” the public would need to be made aware that (*a*) such law exists and the importance of using CRS, (*b*) which CRS are appropriate and meet the law’s safety standards, and (*c*) how to fit children in CRS and CRS in the car. To do so, educational efforts were organised, and a communication strategy was created to reach and educate Filipinos. Coalition members from the media, the Peace and Conflict Journalism Network and Vera Files organised media trainings on road safety and the importance of CRS. Furthermore, media fellowships were made available to train, capacitate, and motivate journalists to write their own road safety pieces “*vetted by experts*.”

 Traffic enforcers would similarly need to be trained on CRS standards and fitting to regulate child safety properly and fairly and, when required, issue the stipulated penalties. Manufacturers, importers, and venders would need to be made aware of the regulations of CRS standards, certification, and expiration; product inspectors would need to be trained on CRS standards; and officials that teach caregivers how to fit CRS (CRS fitters) would also need to be trained. The IRR provided details that tackle these hurdles.^34^

 The implementation of the CSMV Act was scheduled to begin on February 5, 2021. At that time, the police were to start stopping vehicles to ensure the proper use of CRS and issue the prescribed fine for violators. However, on January 29, 2021, Roberto Valera, Land Transport Office (LTO) Deputy Director of Enforcement, held a press conference sharing that they were not yet ready for “*strict enforcement*.” Per domestic NGO and government key informants, implementation activities as stipulated in the IRR were insufficiently executed to expect civilians to be adequately informed about the Act or for officials to be prepared to enforce it. For instance, the Department of Trade and Industry had not yet released a list of approved CRS, few traffic enforcers had completed CRS trainings, fitting stations had not been established, limited fitters had been trained, and too few communications activities had been executed. Instead of strict enforcement, the LTO announced that they would be issuing “*soft enforcement*” for the following three to six months. Soft enforcement included stopping vehicles to issue warnings and share information on CRS, as opposed to issuing penalties.

 Despite the plan to avoid fining CSMV Act offenders, the Act received significant backlash and garnered the majority of its public awareness following the comments of Attorney Clarence V. Guinto, Regional Director of the LTO National Capital Region West. In an interview that aired on a national radio station, DZMM TeleRadyo, on February 1, 2021, Guinto was asked what to do if a 12-year-old child is too big for CRS. Instead of explaining that, per the CSMV Act, children under age 12 who are taller than 150 cm do not need to use CRS, Guinto remarked that the person should “*laki-lakihan mo sasakyan mo*,” which literally translates to “*make your car bigger*,” with the actual connotation being closer to “*get* (or use) *a bigger car*.” However, on social and print media, the statement was understood to mean that Filipinos should “*buy a bigger car*.” This slight misquotation went viral on social media and quickly triggered widespread public backlash. The comment seemingly made “*in jest,*” was perceived by the media as insensitive to Filipinos of limited socioeconomic means, particularly during the hardship of the COVID-19 pandemic, and hence the CSMV Act was labelled as “*anti-poor*” by the media and was opened up to additional criticism unpacked below in the “Naming” sub-section. Guinto later apologised for the inaccurate remark.

 “*Many of the people here in the Philippines resisted the implementation of the law last year, to the point that the Senator JV Ejercito and other advocates of the law were vilified in the media” *(ID 16, Legislative).

####  Act IV: Bureaucratic Uncertainty and Delay

 In the face of this public backlash, President Duterte publicly announced the postponement of the CSMV Act’s implementation on February 2, 2021. Legislative and government stakeholders stressed that this “*deferment*” was not a legal change the President could apply. The House of Representatives, on February 10, 2021, resolved that a new law would need to be passed to officially suspend the implementation of the CSMV Act. Furthermore, the deferment could be considered unnecessary because traffic enforcement was not yet planning to issue fines for CRS violations. Nevertheless, the words of the President were heeded; traffic enforcement would not issue fines before the deferment ends and public apprehension around the Act decreased. However, no clear timeline exists for how long the deferment would last. Some stakeholders falsely believed it would end in mid-August 2021, while others believed that the length of the deferment was tied to the length of the COVID-19 pandemic. “Non-enforcement” implementation activities continued despite this deferment, such as public educational efforts, training law enforcement, and training CRS fitters.

###  Naming: Frames Used to Portray the CSMV Act

 Having established a narrative of the trajectory of the CSMV Act, we now examine how these elements were discursively framed through specific naming practices.

 In the transition from legislation to practice, we identified at least three overarching policy action frames that cast the CSMV Act’s provisions in a negative light. These frames emerged primarily from the key informant interviews where stakeholders reported that members of the public criticized the Act for being “*anti-poor,*” “*unnecessary*,” or a “*strategic political distraction*.” Of them, the idea that the law was “*anti-poor*” was the most common and complex critique of the CSMV Act. The expression “anti-poor” is commonly used in the Philippines. It can be found in academic literature and the media alike, across topics such as the war on drugs, agrarian reform, and COVID-19.^35-40^ The quip seems to be used to refer to any economic strain on Filipinos and accordingly, we have also employed it as a label for any framing related to expense-, financial-, or income-related arguments. While this framing was initially advanced by select legislators during early debates, it only gained traction after the act was established following media coverage of an insensitive remark by a road traffic official (See Act III). This framing was then amplified across social media platforms and, per all 27 key informants, widely adopted by the general public.

 “*You have a lot of pundits [that] are saying this is “anti-poor.” […] That’s the tenor of the conversations that we’ve seen on social media” *(ID 12, Domestic NGO).

 As described in Table 3, we unpack and categorise these anti-poorframes as “country,” “population,” “family,” and “individual” level frames, in addition to an “aggravating exigency” frame. In this way, we demonstrate how frames resonate with different types of constituents, damaging the Act’s prospects on multiple fronts. The country-level frame holds that the Philippines, as a nation, is not financially suited for CRS. At the population-level, as most Filipinos do not own private motor vehicles (PMVs) due to insufficient income, this law does not protect them. The family level frame focuses on the space restrictions that CRS cause when transporting multiple family members, which insinuates the need for larger or additional vehicles. At the individual level, most simply, CRS are considered an added financial burden. Finally, the aggravating exigency frame posits that COVID-19 already financially burdens Filipinos, and the CSMV Act should not add to that incumbrance. In sum, frames that claim that the Act is inappropriate, either because it adds unreasonable expenses or because it excludes those who cannot afford CRS (or the PMVs to necessitate them), classifying the CSMV Act as anti-poor.

**Table 3:** Unpacking the “Anti-Poor” Classification

**Frames**	**Explanation of the Argument**	**Example Quote**
Country level frame	CRS laws are common in and appropriate for HICs. The Philippines is a lower-middle income country and accordingly a CRS law is not appropriate.	*“Child safety seats are thought of as a thing for first world countries*” (ID 01, Domestic NGO).
Population level frame	The majority of the population in the Philippines does not use and cannot afford PMVs. A piece of legislation that does not protect most of the population, discriminates against the safety of non-private vehicle owning families and is hence not appropriate.	*“Look, in our provinces most of the children ride either jeepneys or tricycles, sometimes buses. So, this will not even apply to us. So, it is anti-poor” *(ID 15, Legislative).
Family level frame	In the Philippines it is common to have larger families that travel together. Not as many passengers can fit in a car when children are using CRS. A piece of legislation that is *de facto* obligating households to purchase a larger car to transport their family is an unreasonable expense and is thus not appropriate.	*“I have three kids, but our car can only accommodate two car seats.* […]* What do I do? Do I buy a bigger car?” *(ID 18, Domestic NGO).
Individual level frame	Having a PMV is already very expensive. Adding another layer of expense by legislatively mandating the purchase of a CRS is not appropriate.	*“Car seats really are expensive, especially if you don’t know any other parent using it, you’re going to see it as an unnecessary expense” *(ID 23, Domestic NGO).
Aggravating exigency frame	Amidst the COVID-19 pandemic, many people have faced increased economic hardship. Mandating the extra expense of a CRS is hence not appropriate.	*“That’s because you know the pandemic. A lot of people right now are experiencing economic hardship here in the Philippines because of the lockdowns. So, to them that is additional cost, that is not, let’s say that is not their priority” *(ID 03, Government).

Abbreviations: CRS, child restraint systems; NGOs, non-governmental organizations; HICs, high-income countries; PMV, private motor vehicle.

 The second set of frames used against the CSMV Act can be grouped under the idea that a CRS law is unnecessary. Like the population level anti-poor frame, informants across all sectors reported that many Filipinos understood the CRS law to be unnecessary because few people use PMVs. This frame was predominantly advanced by media commentators and subsequently adopted by members of the public, as reported by our respondents from implementing agencies. Alternatively, the law is reportedly believed to be unnecessary because, like the aggravating exigency anti-poor frame, at the time of implementation, the COVID-19 pandemic was ongoing and children were not permitted to leave the house, and hence did not require CRS. Individuals in metropolitan areas reportedly claimed that CRS are unnecessary because traffic is too heavy to warrant their need. Finally, the law was deemed unnecessary because, in direct contradiction with scientific findings and recommendations, “*nothing is safer than a mother’s arms.*” This frame falsely posits that a parent or caregiver can hold the child in the car to protect them in the case of a crash, and as such, CRS are unnecessary.

 “*We live in a city, and it’s almost always traffic, so, you know, cars don’t go that fast. If ever we do get in a road crash *[without CRS]*, it’s probably not going to be fatal because that’s the speed that we’re going at” *(ID 23, Domestic NGO).

 “*Everyone was baffled because no one was leaving their house *[because of COVID-19 restrictions]*, even children are not allowed. So why is there an urgency to implement the law?”* (ID 16, Legislative).

 “[People thought]* it is safer that the children are embraced by the parents during the travel rather than using CRS” *(ID 22, Government).

 A third frame speaks of strategic political distraction. Informants across the International Organisation, Domestic NGO and Legislative sectors shared that the law is reportedly considered an attempt to divert attention from poor governance, such as that surrounding the pandemic, or the drug war. By introducing road safety legislation, the government was perceived as attempting to demonstrate to the public how they are acting with the protection of their citizens in mind. Stakeholders believed that this “*good press*” (pre-media backlash) was an attempt to mask errors or injustices that occurred under President Duterte’s administration. Along the same lines, the CSMV Act was also understood to be yet another mechanism through which traffic officers can solicit bribes. Traffic enforcement is perceived to be an easy channel for governmental authorities to coerce civilians to pay officers to avoid more costly and inconvenient penalties.

 “*He *[President Duterte]* wanted to have also something that his administration can claim to be a pro-child measure because he was accused of all these extrajudicial killings. With lot of children being killed both in the crossfire of his hard-line policy against drugs” *(ID 26, Domestic NGO).

 The media played a central role in amplifying and legitimising these frames. What began as individual critiques by select officials was transformed through media coverage into dominant narratives that shaped public perception of the Act and opened it up to additional critical frames. As several respondents noted, once these frames became established in media coverage, they became difficult to counter even with evidence-based advocacy.

 CRS and road safety advocates worked to construct frames in opposition to those presented above. They argued that the Bill is pro-poor as it seeks to protect against the high costs of child road safety injuries. The Act counters colonial rhetoric that only HICs’ children should be protected on the roads. Advocates also worked hard in an attempt to frame CRS mandates as a public health issue, as opposed to a road safety one. By centring the health and well-being of children, stakeholders believed the law would receive more support and may have been seen as complementary to COVID-19 public health efforts, as opposed to distracting from them. Finally, to mitigate abuses of power by traffic enforcers, the Philippines has taken preventative measures such as implementing “*bodycams*” that officers cannot turn off. Overall, while CRS advocacy efforts were originally successful in persuading politicians to pass the CSMV Act, public discourse left Filipinos unconvinced. Experts expressed that they felt the push-back against the Act was amplified by pent-up stress and frustration from the COVID-19 pandemic. The negative effect surrounding the pandemic was seemingly transferred to the CSMV Act.

 “*There would be opposition to this policy being anti-poor, but they would explain how this policy is actually pro-poor because it protects people from the social economic impact of road crashes then that debate usually died down”* (ID 21, International Organisation).

 “*We didn’t succeed a lot in developing a lot of public support, but still succeeded with this legislation because of the political support, this is not ideal*” (ID 26, Domestic NGO).

## Discussion

 The CSMV Act in the Philippines passed quickly and efficiently due to a coordinated network of NGOs with philanthropic support who successfully lobbied political actors. Despite minimal legislative resistance and having presidential endorsement, the Act’s implementation stalled following viral comments from a public official that amplified existing “anti-poor” frames and fostered additional criticism of the Act. Intense media backlash during the COVID-19 pandemic, without sufficient public endorsement, placed the law’s enforceability in limbo. This highlights several important policy insights.

 First, the ways in which policies are framed matter. When ideas—in this case, the ideas surrounding the importance or purpose of the CSMV Act—are framed in a particular way, it facilitates a “normative shift” from *statements of fact* to *statements of volition*. Frames work because they harness the irrational power of emotion in activating deeply held social values.^20^ If advocates are not able to manage the narrative and unfavourable policy frames become dominant, a law runs the risk of becoming a “dead letter” – losing its force and authority without being formally abolished.

 The CSMV Act demonstrates that if a policy is framed as punitive rather than protective, especially towards vulnerable groups, the policy’s goals may be undermined. Given the price of CRS and the fines for cases of violations, not to mention the expense of buying a bigger car if that were accurate, the “*anti-poor*” frame seemed to be easily and predictably applied to the CSMV Act.^32^ Financial concerns around public health policies are a frequent negative framing that can jeopardise the success of a bill, as seen in framing research in Kenya on health financing and the United States of America on COVID-19.^41,42^ However, the financial consequences targeting the poorest faction of the population seems largely unique to the Philippines.^35-40^ While the Philippines road safety advocates sought to frame the CSMV Act as a public health measure protecting children and preventing the financial burden of road crashes, the external framing of the Act as punitive, especially for those who are already less well off, dominated the discourse.

 Furthermore, the additional framing of the act in the media as *“unnecessary”* and a *“political distraction”* highlights a potential lack of political legitimacy of the Act. The framing of the law as unnecessary appears to stem from a current lack of cultural acceptability and awareness surrounding CRS in the Philippines.^43^ Incurring doubt around the necessity of a piece of legislation is often detrimental to its success, as seen in the instance of substance use policy, for example.^44^ Moreover, the strategic political distraction frame speaks to apparent public mistrust of government. Without a perceived need for a policy, enacted by a trusted body, the political authority of the Act may be called into question.

 Advocates highlighted the need for CRS legislation given the number of road safety crashes and injuries in the Philippines. CRS use and legislation in the Philippines was an international priority introduced and promoted by global actors at the local NGO level. With the help of funding and technical expertise, local NGOs were able to garner legislative support for policy change. However, the law was enacted with very little public support, which may be linked to insufficient civilian or bottom-up priority-setting. Donor mobilisation and control of resources often shape policy priorities globally.^45,46^ Involvement of grassroots-level actors, public acceptance, or a portrayal of the issue that resonates with external audiences are often considered helpful components of policy prioritization.^46,47^ Though, in this case, funding was used to generate advocacy around CRS in the Philippines that facilitated meaningful legislative dialogue and expert consultation, which allowed for the passage of legislation to save the lives of Filipino children, but without sufficient public ownership, leaving room for a framing backlash.

 Secondly, this case study offers important insights into how framing processes operate throughout a policy’s lifecycle, particularly highlighting the critical role of framing during implementation phases. While much framing research focuses on the stages of policy formulation and agenda-setting, our findings highlight how frames that emerged or solidified during implementation can fundamentally alter a policy’s trajectory even after successful legislative passage. For instance, while recent scholarship on health taxes in LMICs has shown how frames shaped initial policy formulation, our case reveals how frames that emerged *after* legislative success critically influenced implementation outcomes.^48^ Our study contributes to an emerging literature on the relationship between framing and implementation processes. While Nilsen et al and Matland theorise how implementation outcomes are influenced by how policies are understood by the actors that implement them, our findings demonstrate how public framing can override implementation structures even when implementing agencies are committed to the policy.^49,50^

 Moreover, from a theoretical perspective, this case highlights the temporal dimension of framing processes, showing how frames that emerge late in the policy process can retroactively destabilise policies that appeared to have successfully navigated earlier political hurdles. For instance, President Duterte’s decision to defer implementation—though not legally within his authority— shows one such example of a post-hoc destabilisation power. Though we acknowledge that this pattern of constitutional norm-stretching mirrors other Duterte-era policies and suggests broader governance implications beyond this specific case, it remains a stark example of the destabilising potential of framing.^51,52^ The temporal aspect of framing has received insufficient attention in the literature and merits further investigation.

 Third, the CSMV Act stresses how control of the narrative, by the media in particular, is key in garnering popular support. This builds on recent calls, such as those by Cairney and Oliver, for greater attention to political communication and media narratives in shaping implementation environments.^53^ Social and print media are prominent factors for both advocacy/educational efforts and opposition/counter-advocacy.^54-57^ The road safety actors in the Philippines who advanced the passage of the CSMV Act were aware, early on, of the media’s importance to drive public education and support. Despite organised efforts to promote CRS, domestic NGO and legislative stakeholders felt that they were not successful in getting the media’s attention until later when the Act faced a backlash. The LTO member who was understood to say that parents needed to “*buy a bigger car*” was reportedly not trained to speak to the CRS legislation. Following this comment, road safety advocates lost control of the narrative and the CSMV Act became viewed as an oppressive, unnecessary piece of legislation. While studies of COVID-19 framing in the United States demonstrated how political discord shapes public responses to policy, our study shows how a single catalysing event (the official’s “bigger car” comment) can trigger frame transformation that reverberates throughout the implementation process.^42^

 As this study relied on (a qualitative) frame analysis, we were able to outline a rich contextual understanding of the policy trajectory of the CSMV Act; however, the study cannot establish generalizable cause and effect relationships between frames and policy outcomes.^58^ Another limitation of this paper is that relatively few government officials (n = 4) agreed to participate in the study’s key informant interviews (out of n = 11 invited to participate). House of Representatives member Matt Jocabe, who was arguably the CSMV Act’s strongest opponent at the legislative level, was not able to participate in the study due to his recent passing. Furthermore, key informants interviewed did not include members of the general public, nor did the study implement methods that would allow us to quantify how widespread the given frames were among the broader Filipino population. Instead, public perceptions were reported by key actors involved in the Act’s passage and/or implementation. Due to the outsized number of NGOs involved in the passage of the CSMV Act, they were seemingly overrepresented in the sample. However, this reflects the actual composition of stakeholders centrally involved in the policy process, as confirmed through document analysis and consistent with purposive sampling approaches in qualitative research. Due to project and timeline constraints, this paper cannot speak to the impact of the CSMV Act. More research is needed to follow the deferment and to then measure the Act’s impact on CRS use in the Philippines. Finally, this study only focuses on one LMIC, and therefore, readers should be careful when trying to transfer the findings to other LMIC contexts.

## Conclusion

 This study highlights that, with the help of a coordinated coalition and strategic advocacy, the Philippines succeeded in swiftly passing a CRS law that meets UN standards. The law and corresponding IRR provide robust details on how to implement the law to engender and sustain CRS behaviour-change. However, the trajectory of the CSMV Act in the Philippines demonstrates that the passage of legislation is not enough. How policies are framed throughout their social lives matters. Without controlling the narrative of a law and entrenched legislative legitimacy among the public, especially given the political climate surrounding COVID-19, various framings emerged that countered the Act. Negative frames circulating in the media caused President Duterte to defer the enforcement of the CSMV Act. Though legally still binding, with non-enforcement related implementation activities on-going (such as educational efforts), the law is left in a state of limbo with no clear “full implementation” timeline. Urgent action is required to re-frame the public discourse and mobilise public support for CRS use and the CSMV Act, to ensure that the lives of Filipino children are protected by the enforcement of this important piece of legislation.

## Acknowledgements

 This study was made possible by the BIGRS. The authors thank Rhia Muhi and Sophia San Luis for their help in early conceptualization of the study and providing initial assistance with data collection. Also, the authors thank Emily Mendenhall for reviewing the manuscript and providing strategic direction in addition the many experts and advocates interviewed for this study for sharing their time and insights.

## Ethical issues

 The Johns Hopkins Bloomberg School of Public Health Institutional Review Board Office determined this work “Not Human Subjects Research” on March 20, 2020. The National Ethics Committee (NEC), Philippine Council for Health Research and Development, Department of Science and Technology provided ‘Ethical Clearance for the implementation of this study’ effective April 12, 2021, to April 11, 2022. In accordance with NEC protocol, all participants provided written consent to participate in key-informant interviews prior to the interview.

## Conflicts of interest

 Authors declare that they have no conflicts of interest.

## Disclaimers

 All study investigators have been supported by the BIGRS. Though the activities of this Initiative feature in the present analysis, the funder had no role in the collection, analysis, or interpretation of data. We declare no other competing interests.
